# Supplementary material for: Lack of PKCθ Promotes Regenerative Ability of Muscle Stem Cells in Chronic Muscle Injury
Source: Int J Mol Sci. 2020 Jan 31;21(3):932. doi: 10.3390/ijms21030932 (PMC7037041; doi:10.3390/ijms21030932)
Supplement: Supplementary file 1 [file ijms-21-00932-s001.pdf]

Supplementary Materials:

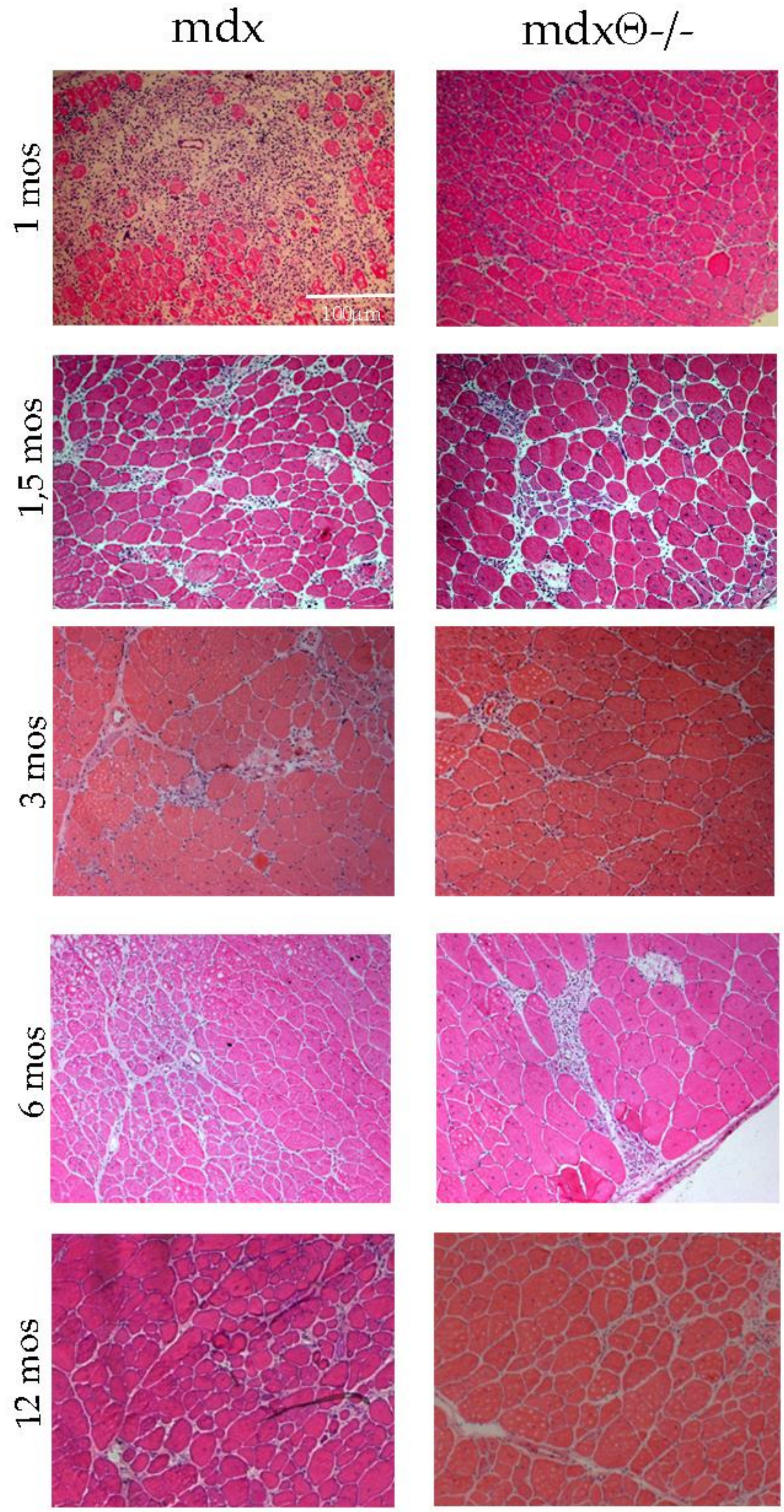

Supplementary Figure 1. Representative images of -H&E staining of cryosections derived from mdx and mdxθ-/- TA muscle at 1.5, 3, 6 and 12 months (n= 4-5/age/genotype).

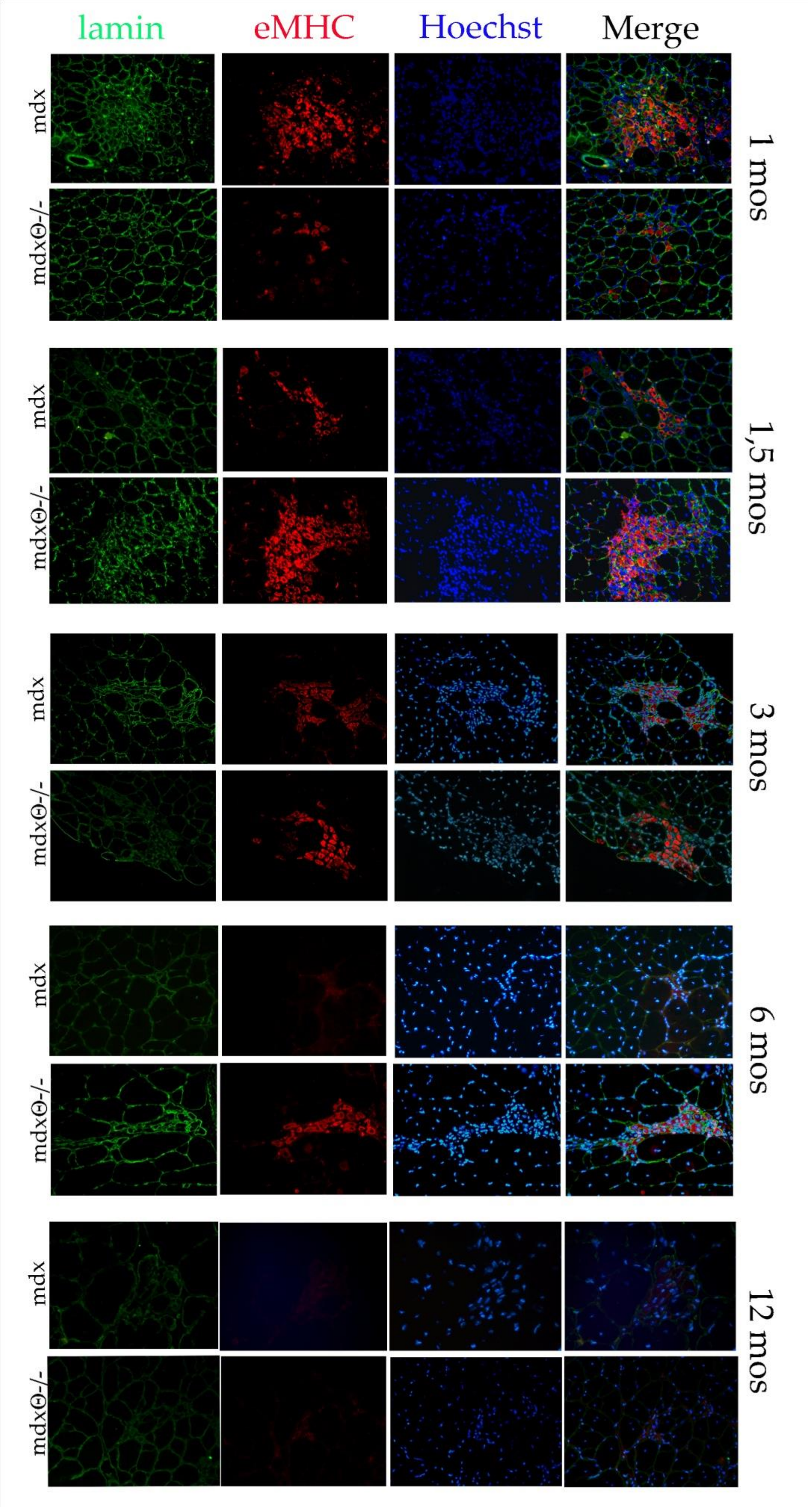

Supplementary Figure 2. Representative images of eMHC (red) and Laminin (green) immunofluorescence staining of TA section from 1, 1.5, 3, 6 and 12 months old mdx and mdxθ-/- mice (n= 4-5/age/genotype). Nuclei were counterstained with Hoechst (blue).

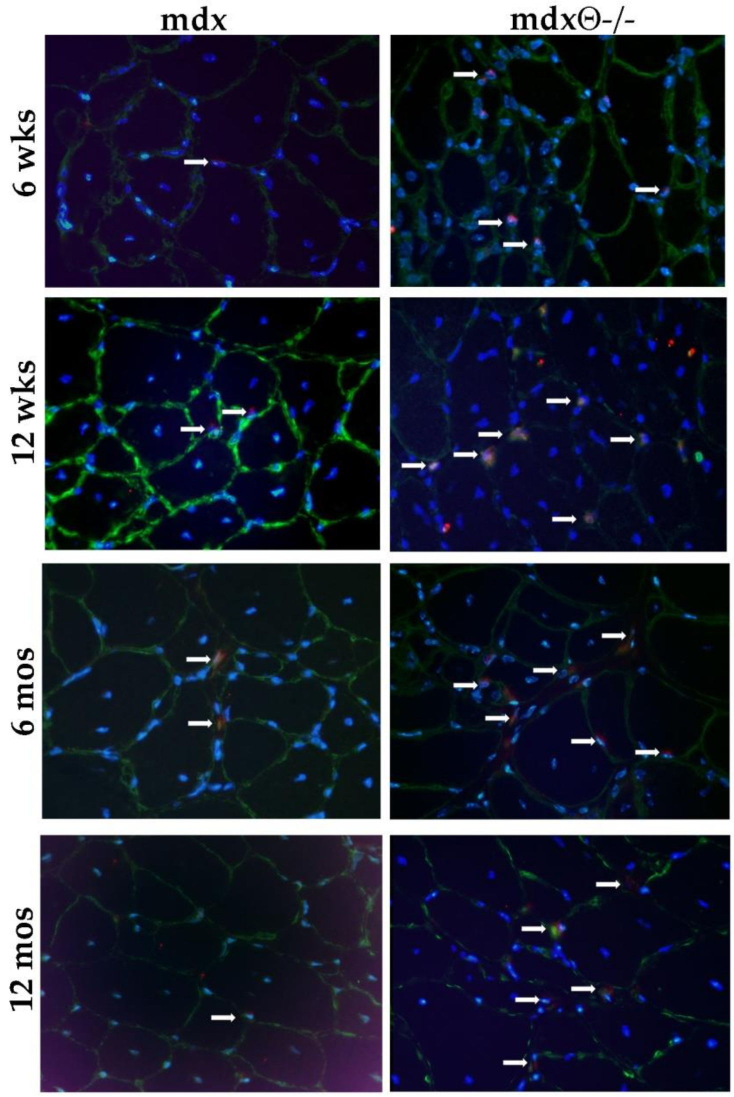

Supplementary Figure 3. Representative images of Pax7 (red) and Laminin (green) immunofluorescence analysis of TA section from 6 and 12 weeks, 6 and 12 months old mdx and mdxθ-/- mice (n=3/genotype/age). Nuclei were counterstained with Hoechst (blue).

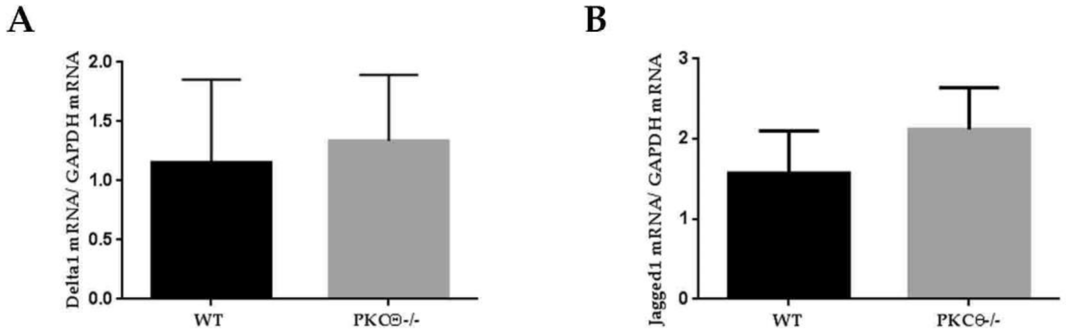

Supplementary Figure 4. qRT-analysis of Delta1(**A)** and Jagged1 (**B**) expression in healthy WT/bl6 and PKCΘ TA (n=5/genotype) muscle. RNA expression level in WT/bl6 and PKCθ-/- was normalized against GAPDH.

**GENE**	**Primer sequences (5′-3′)**
Notch1	
for	GGT CGC AAC TGT GAG AGT GA
rev	TTG CTG GCA CAT TCA TTG AT
Notch2	
for	GCA GGA GCA GGA GGT GAT AG
rev	GCG TTT CTT GGA CTC TCC AG
Notch3	
for	GTC CAG AGG CCA AGA GAC TG
rev	CAG AAG GAG GCC AGC ATA AG
Delta1	
for	CCG GCT GAA GCT ACA GAA AC
rev	GAA AGT CCG CCT TCT TGT TG
Pax7	
for	GTCCCAGTCTTACTGCCCAC
rev	TGTGGACAGGCTCACGTTTT
MyoD	
for	CGACACCGCCTACTACAGTG
rev	GGTGGTGCATCTGCCAAAAG
Numb	
for	CCGGCATGCTCCAATTG
rev	TCTGGCTAAGAGCAGGAAAACC
MyoG	
for	GCATGGAGTTCGGTCCCAA
rev	TATCCTCCACCGTGATGCTG
GAPDH	
for	ACC CAG AAG ACT GTG GAT GG
rev	CAC ATT GGG GGT AGG AAC AC
Jagged1	
for	ATCGTGCTGCCTTTCAGTTT
rev	GGTCACGCGGATCTGATACT

Supplementary TableS1 Primers used for qPCR
